# The Epidemiology of Neurological Complications in Adults With Sickle Cell Disease: A Retrospective Cohort Study

**DOI:** 10.3389/fneur.2021.744118

**Published:** 2021-12-15

**Authors:** Chinedu Maduakor, Vafa Alakbarzade, Yezen Sammaraiee, Angeliki Vakrinou, Alina Corobana, Julia Sikorska, Elizabeth Rhodes, Anthony C. Pereira

**Affiliations:** ^1^Neurology Department, St George's University Hospitals National Health Service Foundation Trust, London, United Kingdom; ^2^Hematology Department, St George's University Hospitals National Health Service Foundation Trust, London, United Kingdom

**Keywords:** sickle cell disease, neurological complications, stroke, prevention of stroke, intracranial vasculopathy

## Abstract

**Introduction:** Risk factors for neurological complications in sickle cell disease differ in the adult and pediatric populations. Here, we focused on neurological complications in adults with sickle cell disease.

**Methods:** Patients were selected using the audit data from the St George's Hospital Red Cell Database. The genotyping, demographics, clinical data, and investigation findings were collected.

**Results:** A total of 303 patients were enrolled in the study: hemoglobin S homozygosity (HbSS) genotype 56%, hemoglobin S and C coinheritance (HbSC) genotype 35%, and hemoglobin S and β-thalassemia coinheritance (HbSβ) thalassemia genotype 9%; the mean age was 38.8 years (±13.5 SD) with 46% males. The most common neurological complication was cerebrovascular disease (*n* = 37, 12%) including those with ischemic stroke (10%), cerebral vasculopathy (3%), and intracranial hemorrhage (1%). Ischemic stroke was common among the HbSS genotype compared with other genotypes (8 vs. 1.6%, *p* = 0.001). Comparing the patients with sickle cell disease who had suffered a stroke to those who had not, there was a higher proportion of intracranial vasculopathy (*p* = 0.001, in particular, Moyamoya) and cognitive dysfunction (*p* < 0.0001).

**Conclusion:** Our cohort supports previous reports that the most common neurological complication in adult sickle cell patients is cerebrovascular disease. Strategies to prevent cerebral vasculopathy and cognitive impairment should be explored.

## Introduction

Sickle cell disease affects about 14,000 people in the United Kingdom (1) and millions worldwide (2). Sickle cell patients report poorer quality of life in comparison with the general population and other chronic non-communicable diseases (3).

Sickle cell disease is an autosomal recessive disease due to hemoglobin S homozygosity (HbSS) or coinheritance with other abnormal hemoglobins including hemoglobin C (HbSC) or β-thalassemia (HbS/ß-thalassemia). There is no consensus about the classification of HbS/ß-thalassemia, but it is usually classified in two types: HbSβ^0^ and HbSβ^plus^ thalassemia (4). Both HbSS and HbSβ^0^ genotypes have severe sickle cell disease (SCD) expression and are usually studied in combination (5). Hemoglobin S homozygosity is less soluble than normal hemoglobin, leading to microcirculation impairment, end-organ ischemia, and necrosis.

Stroke is a serious complication of SCD manifesting in children with HbSS and confirmed abnormal transcranial Dopplers (6). The prevalence of cerebrovascular diseases has been reported as 4% in the HbSS genotype with the lowest incidence of ischemic stroke in those aged 20–29 years and higher in children and older patients (6). Silent cerebral infarcts (detected on brain MRI) are associated with a high risk for overt stroke (7), lower IQ, and cognitive impairment (8, 9). There are associations between large vessel vasculopathy and increased volumes of infarction in children with SCD (10). The first-ever ischemic stroke in adults with SCD, however, has a wider variety of causes and has a higher risk of recurrence than those occurring in children (11). The pathophysiology of stroke in adults with SCD is unclear.

The risk factors for neurological complications differ in adult and pediatric SCD cohorts. Multiple epidemiological studies have shown that the risk factors for ischemic stroke in adult patients include the HbSS genotype, severity of anemia, systolic hypertension, male gender, and increasing age (12). The best-studied clinical risk factors for hemorrhagic stroke are low steady-state hemoglobin and high steady-state leukocyte count (6). Genetic risk factors for stroke in SCD have been an evolving area of investigation with multiple candidate genes evaluated, mostly for ischemic stroke in children, commonly characterized by large-vessel vasculopathy (12). Patients with a Moyamoya pattern of vascularization are pre-disposed to both ischemic and hemorrhagic stroke later in life (13).

For the primary prevention of stroke in children, the Stroke Prevention Trial in Sickle Cell Anemia (STOP) showed that regular blood-transfusion therapy was efficacious (5). Transfusions were later found to reduce the recurrence of silent cerebral infarcts, as shown by the Silent Cerebral Infarct Multi-Center Clinical Trial (SIT) (14). However, there are long-term consequences of chronic transfusions. A transcranial Doppler With Transfusions Changing to Hydroxyurea (TWiTCH) trial revealed that hydroxyurea (or hydroxycarbamide) may be equally effective in the primary prevention of neurological complications including stroke (15). There is some evidence that regular blood transfusion therapy is partially effective for the secondary prevention of strokes in children with HbSS or HbSβ^0^ thalassemia (16–19). However, the best secondary prevention of stroke in adults with SCD is still unclear.

In this study, we aim to analyze: (1) the association of sickle cell genotypes and phenotypes with neurological complications; (2) the risk factors for ischemic stroke in adults with SCD; (3) correlation between cerebrovascular disease and cognitive morbidity; (4) impact of transfusion and hydroxycarbamide on the stroke incidence in adults with SCD.

## Materials and Methods

We performed a retrospective review of consecutive, SCD cases from the St George's Hospital Red Cell Database reviewed from 2016 to 2019. This is the main center for stroke, neurology, and hematology referrals in South West London. We included: (1) patients with SCD defined as HbSS, HbSC, HbSβ^0^, and HbSβ^plus^ thalassemia genotypes confirmed with hemoglobin analysis and other confirmatory documentation of phenotype; (2) patients ≥ 18 years; (3) patients with any infarct status including none, childhood or adult-onset, silent cerebral infarcts or overt stroke; (4) patients with any therapy modality for stroke prevention. Patients with no available clinical documentation were excluded.

The demographics and clinical data were collated by five clinicians (CM, YS, AV, AC, JS, and ER) using a standardized proforma/template containing the variables of interest. The clinical data included demographics, vascular risk factors, medical comorbidity, and different investigations findings including CT and MRI head, spine, and intra-cranial MR angiograms. All available MRI reports and reasons for referrals were viewed. Brain MRI was predominantly arranged due to the neurological or cognitive symptoms. Psychometric testing measures, transcranial, and carotid Doppler data were not available for most of the cases and therefore, could not be analyzed further.

Statistical analysis was performed in SPSS (V.23; SPSS Inc., Chicago, Illinois, USA). Depending on the normality of distribution as assessed by the Kolmogorov-Smirnov test, and continuous variables were compared using the *t*-test for independent samples. Categorical variables were analyzed as frequency and percentage, and the differences among these variables were assessed by the Chi-squared test. For the univariate correlation analysis, Pearson's *R* was used. Logistic regression analysis was used to assess the correlation of cerebrovascular disease, including ischemic symptomatic or silent stroke, hemorrhagic stroke, and vasculopathy, with cognitive impairment independent of other covariates; and correlation of ischemic symptomatic and silent stroke with cerebral vasculopathy independent of other covariates. The level of significance for these descriptive comparisons was established at 0.05 for two-sided hypothesis testing.

## Results

A total of 303 patients met the criteria for inclusion and subsequent analysis. The mean age of the patient cohort was 38.8 years (±13.5 SD) with 46% male. Genotyping revealed 170 (56%) HbSS (including nine cases with HbSβ^0^ genotype), 107 (35%) HbSC, and 26 (9%) HbS/ß-thalassemia genotype. Sickle cell cases with HbSS occurred more in younger and male patients compared with other genotypes (Table 1). Sickle pain crisis and acute chest syndrome were the most common complication, with values of 42 and 12.5%, respectively. The most frequent chronic complications were renal, osteonecrosis, retinopathy, pulmonary hypertension, or gallstones. The list of other comorbidities is in Supplementary Table 1.

**Table 1:** Demographics and common phenotype of sickle cell sub-types.

**Characteristics**	**All *N* = 303**	**Sickle cell sub-types**		***P*** **-value**
		**HbSS^†^** ***N*** **= 170**	**HbSC and HbS/ß-thalassemia *N* = 133**	
Age^*^	38.8 (13.5)	36 (11)	42 (14)	**<0.001**
Male sex	139 (46)	89 (52)	50 (38)	**0.01**
Acute chest syndrome	39 (12.5)	22 (13)	17 (13)	0.96
Sickle pain crisis	127 (42)	87 (51)	40 (30)	**<0.001**
Priapism	8 (2.6)	4 (2.4)	4 (3)	0.72
Nephropathy	32 (10.5)	16 (9.4)	16 (12)	0.46
Osteonecrosis^**^	29 (9.5)	15 (8.8)	14 (10.5)	0.61
Retinopathy^***^	28 (9)	4 (2.4)	24 (18)	**<0.001**
Pulmonary hypertension	16 (5.2)	12 (7)	4 (3)	0.11
Gallstones^****^	14 (4.6)	8 (4.7)	6 (4.5)	0.93

* *Mean standard deviation of years.*

** *Those resulting in fractures, deformity, and vertebral collapse requiring chronic analgesia and surgical interventions.*

*** *Those with proliferative retinopathy.*

**** *Those necessitating cholecystectomies.*

† *Also includes nine cases of HbS/ß^plus^ thalassemia genotype. Bold values stands for statistically significance*.

The most common neurological complication was cerebrovascular disease, *n* = 37 (12%). The median of conventional vascular risk factors for the sickle cell cohort was 0 (0–2 IQR). There was no significant difference in the proportion of vascular risk factors among the different sub-types except hypertension (Table 2). Among the patients, 37% (60/161) with HbSS had head MRI compared with 44% (4/9) with HbS/ß^0^ thalassemia, 19% (5/26) with HbS/ß^plus^ thalassemia, and 29% (31/107) with HBSC genotype. Additionally, 14% (24/161) of the patients with HbSS had an ischemic stroke (including both symptomatic and silent infarcts) compared with 11% (1/9) with HbS/ß^0^ thalassemia, 7.6% (2/26) with HbS/ß^plus^ thalassemia, and 2.8% (3/107) with HBSC genotype. Four percent (7/161) of the patients with HbSS had a silent stroke compared with none in HbS/ß^0^ and HbS/ß^plus^ thalassemia and 1.9% (2/107) with HBSC genotype. A significant proportion of patients with HbSS cases suffered from ischemic stroke (8%) compared with HbSC, HbS/ß^0^ thalassemia, and HbS/ß^plus^ thalassemia (1.6%). The common cause of ischemic stroke was large vessel occlusion (2.9%) followed by undetermined etiology (2%). There was no significant difference between the different stroke sub-types. Intracranial vasculopathy affected predominantly those with HbSS genotype compared with HbSC and HbS/ß-thalassemia (6% vs. 0) (Table 2).

**Table 2:** Cerebrovascular disease risk factors and classification of sickle sub-types.

**Characteristics**	**All *N* = 303**	**Sickle cell sub-types**		***P*** **-value**
		**HbSS^†^** ***N*** **= 170**	**HbSC and HbS/ß-thalassemia *N* = 133**	
Hypertension	34 (11)	11 (6.5)	23 (17)	**0.003**
Diabetes mellitus	10 (3)	3 (1.8)	7 (5.3)	0.08
Hyperlipidaemia	7 (2)	3 (1.8)	4 (3)	0.47
Smoking	5 (1.6)	2 (1.2)	3 (2.3)	0.46
Ischemic heart disease	2 (0.6)	1 (0.6)	1 (0.8)	0.86
Atrial fibrillation	2 (0.6)	1 (0.6)	1 (0.8)	0.86
Patent foramen ovale	1 (0.3)	1 (0.6)	0	1
Other cardiac disease	3 (1)	1 (0.6)	2 (1.5)	0.42
Ischemic stroke	30 (10)	25 (15)	5 (3.8)	**0.001**
Overt stroke	21 (6.9)	18 (10.6)	3 (2.3)	**0.004**
Silent stroke	9 (2.9)	7 (4)	2 (1.5)	0.18
Childhood stroke	4 (1.3)	4 (2.4)	0	0.13
Small vessel disease	5 (1.6)	3 (1.8)	2 (1.5)	0.85
Large vessel occlusion	9 (2.9)	8 (4.7)	1 (0.8)	**0.044**
Undetermined	6 (2)	6 (3.5)	0	**0.036**
Intracranial hemorrhage	3 (1)	3 (1.8)	0	0.25
Intracranial vasculopathy	10 (3)	10 (6)	0	**0.003**
Moyamoya	3 (1)	3 (1.8)	0	0.25
Aneurysm	6 (2)	6 (3.5)	0	**0.03**
Arteriovenous malformation or fistula	1 (0.3)	1 (0.6)	0	1

† *Also includes nine cases of HbS/ßplus thalassemia genotype. Bold values stands for statistically significance*.

A significant proportion of all patients with SCD and ischemic stroke had hyperlipidemia and ischemic heart disease compared with those without ischemic stroke, *p* < 0.0001 and *p* = 0.01, respectively (Table 3). Patients with SCD and ischemic stroke had a higher proportion of intracranial vasculopathy in particular Moyamoya compared with those without ischemic stroke, *p* = 0.001 (Table 3). Cognitive impairment was significantly frequent in patients with SCD and ischemic stroke compared with those without ischemic stroke, *p* < 0.0001 (Table 3).

**Table 3:** Ischemic stroke and cognitive impairment in the sickle cell disease cohort.

**Characteristics**	**SCD with Ischemic stroke** ***N*** **= 30**	**SCD without Ischemic stroke** ***N*** **= 273**	***P*** **-value**
Age	41.4 (18)	38.5 (12)	0.28
Male sex	17 (57)	122 (45)	0.21
Hypertension	2 (6.7)	32 (12)	0.40
Diabetes mellitus	1 (3.3)	9 (3.3)	0.99
Hyperlipidaemia	5 (16.7)	2 (0.7)	**<0.001**
Smoking	1 (3.3)	4 (1.5)	0.44
Ischemic heart disease	2 (6.7)	0	**0.01**
Atrial fibrillation	0	2 (0.7)	1
HbSS genotype	24 (80)	137 (50)	**0.001**
HbSC genotype	3 (10)	104 (38)	**0.002**
HbS/ß^plus^ thalassemia genotype	2 (6.7)	24 (8.8)	0.69
HbS/ß^0^ thalassemia genotype	1 (3.3)	8 (3)	1
Intracranial vasculopathy	3 (10)	7 (2.6)	**0.03**
Moyamoya	2 (6.7)	1 (0.4)	**0.001**
Aneurysm	1 (3.3)	5 (1.8)	0.57
Arteriovenous malformation and fistula	0	1 (0.4)	1
Cognitive dysfunction	4 (13)	3 (1)	**<0.001**

*Bold values stands for statistically significance*.

Cerebrovascular disease was associated with cognitive impairment (*r* = 0.211, *p* < 0.0001) on univariate analysis and when controlling for age, gender, smoking history, presence of hypertension, hyperlipidemia, diabetes mellitus, obstructive sleep apnea, and mental health disease on logistic regression analysis (*p* = 0.04). Similarly, ischemic stroke was associated with cerebral vasculopathy (*r* = 0.24, *p* = 0.03) on the univariate analysis but was affected by age, gender, smoking, or hypertension on the logistic regression analysis (*p* = 0.057).

In patients with SCD, a significant proportion was on a “disease modifying” treatment (55%) (Table 4). Seventy percent of patients with SCD and ischemic stroke had previous exchange transfusion(s) (with or without Hydroxycarbamide) compared with 43% of those without ischemic stroke. Two cases developed iron overload and one required Deferasirox. There was no significant association between SCD therapy and cognitive impairment or intracranial vasculopathy.

**Table 4:** Stroke prevention in sickle cell disease.

**Characteristics**	**SCD with Ischemic stroke *N* = 30**	**SCD without Ischemic stroke** ***N*** **= 273**	***P-*** **value**
Transfusion	20 (67)	109 (40)	**0.005**
Hydroxycarbamide	2 (7)	22 (8)	0.77
Hydroxycarbamide and transfusion	1 (3)	8 (3)	0.90
No SCD treatment	7 (23)	134 (49)	**0.007**

*One patient had Deferasirox in the SCD with ischemic stroke group. Bold values stands for statistically significance. Bold values stands for statistically significance*.

The second most common neurological comorbidity in adult patients with SCD were neuropsychological conditions (4.2%) including anxiety, depression, psychosis, schizophrenia, suicidal ideation, non-epileptic attacks, and chronic fatigue (Table 5). Additionally, 4.5% of adults with SCD had peripheral neuropathy (axonal or demyelinating) and/or radiculopathy (predominantly due to spinal degenerative disease) (Table 5).

**Table 5:** Neurological comorbidities in the sickle sub-types.

**Characteristics**	**Sickle cell sub-types**		***P*** **-value**
	**HbSS^†^** ***N*** **= 170**	**HbSC and HbS/ß-thalassemia** ***N*** **= 133**	
Neuropathy and/or radiculopathy^*^	0	6 (4.5)	**0.006**
Multiple sclerosis	1 (0.6)	1 (0.8)	0.86
Movement disorders	0	2 (1.5)	0.19
Mental health disease	2 (1.2)	11 (8.3)	**0.002**
Anxiety and/or depression	0	8 (6)	**0.001**

* *This group did not include the patients with sickle cell-related limbs or back pain.*

† *Also includes nine cases of HbS/ß^plus^ thalassemia genotype. Bold values stands for statistically significance*.

## Discussion

Our single-center retrospective study, primarily aiming to investigate the distribution of neurological phenotypes across the adult SCD cohort, revealed that cerebrovascular disease was the most common neurological comorbidity (12%). This is in keeping with studies conducted in pediatric SCD cohorts with the frequency of stroke ranging from 2.9 to 16.9% (20–22). However, the frequency of stroke was higher in our adult SCD cohort compared with previous studies that included adult SCD cases (under 3.9%) (23). Firstly, a lower prevalence of stroke in adult SCD may reflect the decreased survival of children and adolescents with stroke in developing countries (24). Secondly, we included cerebral vasculopathy in the cerebrovascular disease group; that may account for the difference. Thirdly, the frequency of stroke increased with age in our cohort; this was not statistically significant.

Similar to previous studies (6, 12, 25), HbSS genotype was the biggest risk factor for cerebrovascular disease. There are limited studies on the risk factors for stroke in the adult SCD cohorts (12). The higher prevalence risk factors in our stroke cohort were hyperlipidemia (*p* < 0.0001) and ischemic heart disease (*p* = 0.01). Interestingly, hypertension prevalence in our cohort was 11% (Table 2). It is possible that the early use of the angiotensin-converting enzyme inhibitors for proteinuria and the relatively younger age of the cohort yielded a lower proportion of hypertension. Compared with the whole group, patients with HBSS disease had a higher risk of stroke or vasculopathy but lower individual conventional risk factors, suggesting that SCD itself was the major risk. This suggests a role for primary prevention.

Our SCD cohort, in particular those with ischemic stroke, had a higher proportion of intracranial vasculopathy. This was in keeping with higher reported rates of aneurysm formation in patients with SCD than in the general population (>1% in children and 10% in adults) and thought to be secondary to chronic high flow status, change in circulation patterns, and underlying vessel wall damage (26). More patients with an HbSS genotype had intracranial vasculopathy compared with other genotypes (*p* = 0.003).

Cerebrovascular disease in our SCD cohort was significantly associated with cognitive impairment. Children with silent infarcts have been shown to perform significantly lower on neuropsychological tests (27). Similarly, compared with healthy controls, adults with SCD had poorer cognitive performance, which was associated with anemia and age (28). Interestingly, cerebrovascular disease affected cognitive function in our cohort independent of age and other risk factors. Prevention of cerebrovascular disease in SCD is important because neurocognitive dysfunction has been implicated as an important contributor to the poor social, economic, and quality-of-life factors reported in adult SCD (29–31).

A substantial proportion of our SCD cohort with ischemic stroke had regular blood-transfusion therapy compared with those without ischemic stroke (66 vs. 40%). However, the incidence of stroke has been shown to decrease in patients receiving chronic transfusion (32). The role of chronic transfusion for the prevention of recurrent events has not been defined in patients suffering their initial stroke in adulthood. Only 8% (24/303) of patients with sickle cell were treated with hydroxycarbamide monotherapy (Table 4). Our cohort collection and analysis pre-dates the British Society of Hematology 2018 recommendations which state that patients who have been started on chronic transfusion therapy for primary prevention during childhood should be assessed by an expert in SCD at the transition to adult care to discuss the risks and benefits of ongoing transfusion, and should be offered a continuation of transfusion therapy or hydroxycarbamide if they have had a previous abnormal transcranial Doppler that has normalized and there is no evidence of vasculopathy (33). Those who have been started on hydroxycarbamide for primary stroke prevention during childhood should be offered ongoing hydroxycarbamide therapy after the transition to the adult service. However, hydroxycarbamide adherence is suboptimal in young people with SCD (34–36).

Anxiety and depression were the second most common neurological comorbidities in our cohort (2.6%). Studies focusing on children and adolescents with SCD suggest greater risks for psychosocial difficulties, depressive, anxiety symptoms, and SCD-related stigma (37–39). The identification of these risks is important and may influence treatment outcomes (40). Moreover, SCD-related stigma and pain interference in adolescents hospitalized for SCD pain, as these factors may influence treatment outcomes.

Peripheral nervous system involvement was rare in our SCD cohort, similar to previous reports. Reports of patients with SCD who developed acute mononeuropathy multiplex in the setting of sickle cell pain crisis suggest a multifocal nerve disorder resulting from ischemia caused by a sickle cell vaso-occlusive crisis (41). Multiple sclerosis was also rare in our adult SCD cohort, less than the UK general population.

A strength of our study includes the real-world comprehensive assessment of adults with SCD and stroke at the main center in South West London with vascular neurology and hematology specialists for referrals, data collection, and re-analysis thereby limiting misinterpretation of the data and laying foundations for future prospective studies. To our knowledge, this is one of the largest adult SCD cohorts studied aiming to investigate the distribution of neurological phenotypes.

Our data should be interpreted with some caution due to the limitations of the study. These include retrospective bias inherent to the study design, underreported symptoms such as headache and sickle pain crises among HbSS, small sample size, and exclusion of transcranial Doppler and psychometric testing measures due to significant missing data. Physicians rely on patients to provide a history of vaso-occlusive crises, often during peak times of pain when the patient is distressed and seeking medical attention. Therefore, at-home vaso-occlusive crises are underreported. The Evaluation of Longitudinal Pain Study in Sickle Cell Disease (ELIPSIS) trial has recently shown the feasibility of accurately monitoring out-of-hospital pain by using patient-reported vaso-occlusive crisis days as potential endpoints for clinical trials in SCD (42). The utilization of the quantifiable biomarker in future trials has the potential to improve this bias. It is not our routine practice to perform neuro-imaging or cognitive screening in asymptomatic adults with SCD. Therefore, only patients with neuro-cognitive manifestations in our study underwent CT or MRI brain and/or full cognitive assessment. However, this may lead to the under-report of silent infarcts or subclinical cognitive impairment. Neuro-imaging and cognitive screening of the larger cohort with asymptomatic SCD can further improve our understanding of the silent infarcts or subclinical cognitive impairment on the quality of life.

In conclusion, our findings provide additional evidence that the most common neurological complication of adult SCD is cerebrovascular disease. This is associated with other symptoms including cognitive impairment which in turn has its own morbidity. It will be possible to better judge the importance of the findings when the large prospective studies on adult stroke in SCD and primary prevention strategies are developed and trialed.

## Data Availability Statement

The raw data supporting the conclusions of this article will be made available by the authors, without undue reservation.

## Ethics Statement

Ethical review and approval was not required for the study on human participants in accordance with the local legislation and institutional requirements. Written informed consent for participation was not required for this study in accordance with the national legislation and the institutional requirements.

## Author Contributions

AP and ER conceived the study. CM, YS, AV, AC, JS, and ER collected data. AP, CM, and VA oversaw the statistical analysis plan, conducted the statistical analysis, and contributed to data interpretation. AP and CM contributed to data quality assurance and data quality analysis. CM and VA drafted the initial manuscript and AC, AV, AP, ER, JS, and YS critically revised the manuscript. All authors gave final approval for publication.

## Conflict of Interest

The authors declare that the research was conducted in the absence of any commercial or financial relationships that could be construed as a potential conflict of interest.

## Publisher's Note

All claims expressed in this article are solely those of the authors and do not necessarily represent those of their affiliated organizations, or those of the publisher, the editors and the reviewers. Any product that may be evaluated in this article, or claim that may be made by its manufacturer, is not guaranteed or endorsed by the publisher.

## Abbreviations

HbS/ß-thalassemia: Hemoglobin S and β-thalassemia coinheritance
HbSC: Hemoglobin S and C coinheritance
HbSS: Hemoglobin S homozygosity
MRI: Magnetic resonance imaging
SCD: Sickle Cell Disease
SIT: The Silent Cerebral Infarct Multi-Center Clinical Trial
STOP: The Stroke Prevention Trial in Sickle Cell Anemia
TWiTCH: TCD With Transfusions Changing to Hydroxyurea.
