# Supplementary material for: The Epidemiology of Neurological Complications in Adults With Sickle Cell Disease: A Retrospective Cohort Study
Source: Front Neurol. 2021 Dec 15;12:744118. doi: 10.3389/fneur.2021.744118 (PMC8714798; doi:10.3389/fneur.2021.744118)
Supplement: Supplementary file 1 [file Table_1.pdf]

Supplementary Material

Supplementary Table

		Sickle cell sub-types		
**Characteristics**	**All** *N***=303**	**HbSS** *N***=170**	**HbSC & HbS/ß-thalassemia** *N***=133**	***P*-value**
Autoimmune disease	14 (4.6)	8 (2.6)	6 (1.9)	0.93
Sarcoidosis	4 (1.3)	4 (1.3)	0	0.13
Rheumatoid arthritis	4 (1.3)	2 (0.6)	2 (0.6)	0.80
Lupus	4 (1.3)	2 (0.6)	2 (0.6)	0.80
Infections	12 (3.9)	7 (2.3)	5 (1.6)	0.87
Other hematological comorbidities	12 (3.9)	1 (0.3)	11 (4)	**<0.001**
Splenomegaly & thrombocytopenia	6 (1.9)	0	6 (1.9)	0.87
Gastroenterological comorbidities*	11 (4)	3 (1)	8 (2.6)	**0.04**
Osteoarthritis	5 (1.6)	0	5 (1.6)	**0.01**
Endocrinological comorbidities*	5 (1.6)	1 (0.3)	4 (1.3)	0.10
Deafness	4 (1.3)	2 (0.6)	2 (0.6)	0.80
Cataracts	3 (1)	0	3 (1)	0.08
Glaucoma	2 (0.6)	0	2 (0.6)	0.19
Vitreous haemorrhage	3 (1)	0	3 (1)	0.08
Obstructive sleep apnoea	5 (1.6)	5 (1.6)	0	0.06

* excluding gallstones. Percentages in the table are out of the whole group.
